# Identification of temporal condition patterns associated with pediatric obesity incidence using sequence mining and big data

**DOI:** 10.1038/s41366-020-0614-7

**Published:** 2020-06-03

**Authors:** Elizabeth A. Campbell, Ting Qian, Jeffrey M. Miller, Ellen J. Bass, Aaron J. Masino

**Affiliations:** 1 Department of Information Science, College of Computing and Informatics, Drexel University, Philadelphia, PA USA; 2 Department of Biomedical and Health Informatics, Children’s Hospital of Philadelphia, Philadelphia, PA USA; 3 Department of Psychology, Princeton University, Princeton, NJ USA; 4 Department of Health Systems and Sciences Research, College of Nursing and Health Professions, Philadelphia, PA USA; 5 Department of Anesthesiology and Critical Care, Perelman School of Medicine at the University of Pennsylvania, Philadelphia, PA USA

**Keywords:** Obesity, Risk factors, Paediatrics

## Abstract

**Background:**

Electronic health records (EHRs) are potentially important components in addressing pediatric obesity in clinical settings and at the population level. This work aims to identify temporal condition patterns surrounding obesity incidence in a large pediatric population that may inform clinical care and childhood obesity policy and prevention efforts.

**Methods:**

EHR data from healthcare visits with an initial record of obesity incidence (index visit) from 2009 through 2016 at the Children’s Hospital of Philadelphia, and visits immediately before (pre-index) and after (post-index), were compared with a matched control population of patients with a healthy weight to characterize the prevalence of common diagnoses and condition trajectories. The study population consisted of 49,694 patients with pediatric obesity and their corresponding matched controls. The SPADE algorithm was used to identify common temporal condition patterns in the case population. McNemar’s test was used to assess the statistical significance of pattern prevalence differences between the case and control populations.

**Results:**

SPADE identified 163 condition patterns that were present in at least 1% of cases; 80 were significantly more common among cases and 45 were significantly more common among controls (*p* < 0.05). Asthma and allergic rhinitis were strongly associated with childhood obesity incidence, particularly during the pre-index and index visits. Seven conditions were commonly diagnosed for cases exclusively during pre-index visits, including ear, nose, and throat disorders and gastroenteritis.

**Conclusions:**

The novel application of SPADE on a large retrospective dataset revealed temporally dependent condition associations with obesity incidence. Allergic rhinitis and asthma had a particularly high prevalence during pre-index visits. These conditions, along with those exclusively observed during pre-index visits, may represent signals of future obesity. While causation cannot be inferred from these associations, the temporal condition patterns identified here represent hypotheses that can be investigated to determine causal relationships in future obesity research.

## Introduction

Childhood obesity is a major public health issue in the United States. In 2016, ~35 percent of children and adolescents, ages 2–19 years, were overweight (age- and sex-specific body mass index (BMI) greater than or equal to the 85th percentile per Centers for Disease Control and Prevention (CDC) growth charts) [1] or obese (age- and sex-specific BMI greater than or equal to the 95th percentile per CDC growth charts) [1]; approximately half of these children were obese [2]. Children with obesity have elevated risks of developing numerous comorbidities including diabetes, hypertension, sleep apnea, and psychological issues in childhood and later in life [3–5].

Electronic health records (EHRs) have the potential to support childhood obesity diagnosis, treatment, and surveillance at the clinical [6] and population levels [7]. EHR-derived data support the surveillance of obesity and associated comorbidities such as diabetes and asthma [8]. The data are useful for generating large, diverse cohorts for population studies [9] and can be combined with community-level data on environmental factors associated with unhealthy BMI for comprehensive child obesity studies [10].

Prior research has addressed the use of EHRs for obesity diagnosis and quality improvement in clinical settings [11], for prevalence and demographic estimates of childhood obesity and associated comorbidities [8, 12, 13], and in conjunction with other sources of data to study environmental influences on childhood obesity [10]. However, there is limited research on the temporal dependency of conditions associated with childhood obesity incidence. Knowledge of such temporal condition patterns is important because it may signal impending obesity or conditions likely to follow obesity incidence which could support care providers and health policy development. This study’s objective was to identify temporally ordered condition patterns surrounding childhood obesity incidence. Specifically, we sought to identify sequences of conditions recorded in the EHR for healthcare visits immediately before, during, and after the visit in which an obese BMI was first recorded. This work presents the novel application of a sequence mining algorithm, SPADE, to a large retrospective cohort to identify common condition trajectories surrounding pediatric obesity incidence. The approach is designed to:

1. Identify common temporal condition sequences surrounding pediatric obesity incidence and conditions that are more prevalent before or after incidence.
2. Determine if these condition patterns occur at a statistically significant different prevalence in patients with obesity as compared with similarly matched patients with healthy BMIs.

## Materials and methods

### Setting

We implemented a retrospective, matched case control study using a dataset derived from the Pediatric Big Data (PBD) resource at the Children’s Hospital of Philadelphia (CHOP) (a pediatric tertiary academic medical center). The PBD resource includes clinical data collected from CHOP, the CHOP Care Network (a primary care network of over 30 sites), and CHOP Specialty Care and Surgical Centers. Both clinical and non-clinical observations (as defined by Observational Health Data Sciences and Informatics (OHDSI) condition domain standards) from a patient’s EHR are included in the PBD database [14]. The PBD resource contains health-related information, including demographic, encounter, medication, procedure, and measurement (e.g., vital signs, laboratory results) elements for a large, unselected population of children seen in the CHOP healthcare network. Non-study personnel extracted all data from the EHR and removed protected health information identifiers, with the exception of dates, prior to transfer to the study database. Date information was removed from the analysis dataset as described below. The CHOP Institutional Review Board approved this study and waived the requirement for consent.

### Inclusion criteria

This analysis used the CDC definition of childhood obesity (BMI *z*-score at or above the 95th percentile for age and sex) [15, 16]. Patients had at least one obesity measurement during a CHOP primary care visit and at least one visit prior to the first obesity measurement where an obese BMI was not recorded.

Data in the PBD resource are indexed by patient and visit (i.e., encounter). For purposes of this analysis, we consider a record to refer to a single patient visit and all data associated with that visit. Records that were not generated at a CHOP primary care site were excluded. Negative height, weight, and age values were removed from individual records. Height and weight measurements obtained on the same date were matched and used to calculate BMI. For records with duplicate entries of the same height or weight value recorded on the same date, only a single BMI value was used for that date. If there were different height or weight values on the same day, the most recent values recorded were used. The BMI *z*-scores were centrally calculated in this analysis. The same definition of obesity was used across study sites for the entire study period.

Patients must have been 2–18 years old during the index visit (the first time a patient is recorded with an obese BMI). Per CDC guidelines [17], biologically implausible height, weight, and BMI values were excluded from patient records, and we required that the BMI *z*-score (at or above 1.6449) during the index visit must have been biologically plausible; <1% of patients had biologically implausible BMIs at the time their BMI was first documented as obese (*n* = 303). Index visits coded as inpatient stays, ambulatory visits, or emergency department visits (face-to-face encounters) were included. The visit must have occurred between January 1, 2009 and December 31, 2016. Patients without a visit prior to the index visit were excluded.

**Fig. 1:**
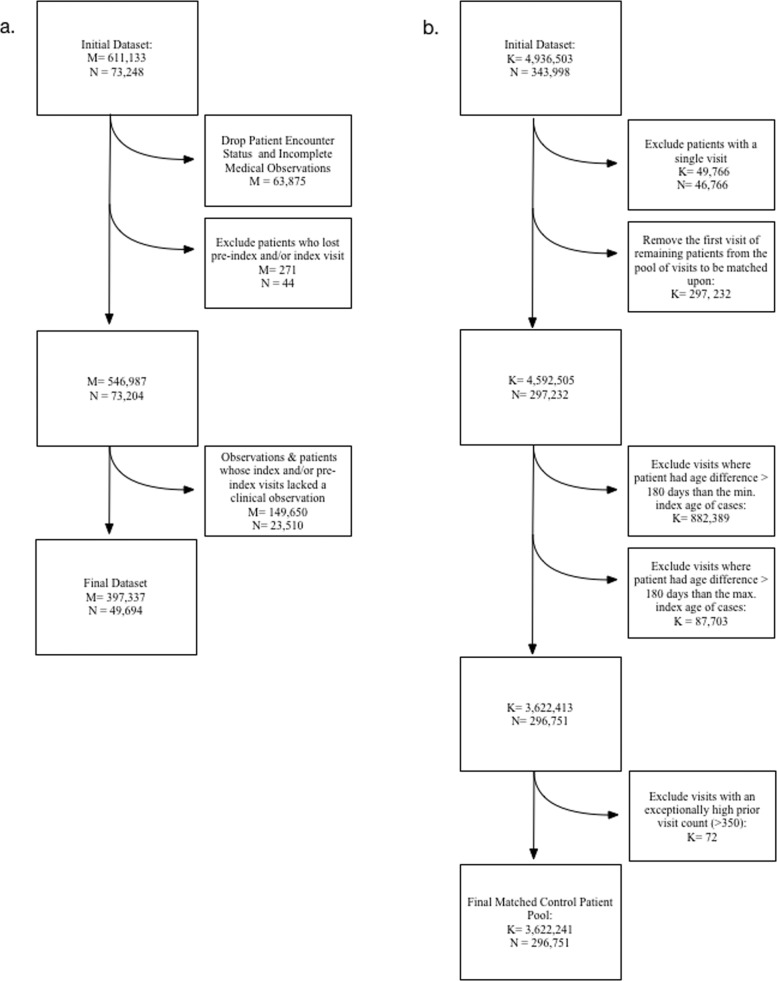
Study Flow Diagram. Study flow diagrams illustrating (**a**) data cleaning and processing of EHR data for the case population and (**b**) data acquisition process of visit data for potential matched controls. In **a**, *M* represents the number of observations (diagnostic codes) and *N* represents the number of patients. In **b**, *K* represents the number of visits and *N* represents the number of patients. In **a**, all patients had an index visit between January 1, 2009 and December 31, 2016 that was a face-to-face encounter and included height and weight measures that yielded a biologically plausible BMI. All patients had a pre-index visit. In **b**, all patients had BMI measurements that only fell between the 5th and 84th percentile based on height and weight measurements recorded from January 1, 2009 and December 31, 2016. Note, the *Patient Encounter Status* concept is used to indicate an encounter without medical findings. Use of this concept was not included in our analysis as it does not provide diagnostic information.

The case patient data selection and cleaning processes are summarized in Fig. 1a. Data from the latest prior visit and the earliest proceeding visit for each patient were selected as a patient’s pre- and post-index visits if applicable. The pre-index and post-index visits were not necessarily face-to-face encounters, but must have occurred between January 1, 2005 and December 31, 2017. All patient visits were required to have had at least one recorded clinical finding per OHDSI condition domain standards (for example, an anemia screening is a non-clinical observation while an anemia diagnosis is a clinical observation) [14]. Condition observations are represented by ICD-9-CM and ICD-10-CM concepts [18, 19]. We note that once the pre-index, index, and post-index visits, and their corresponding separation in number of days, are identified and labeled as such, date information is not required for the subsequent analysis.

The final dataset was comprised of 397,337 clinical and non-clinical observations for 49,694 patients; 33.4% were recorded during a pre-index visit (*n* = 132,786), 40.8% were recorded during an index visit (*n* = 161,944), and 25.8% were recorded during a post-index visit (*n* = 102,607). Approximately 2/3 of patients (*n* = 33,839) had a non-obese BMI measurement in at least one visit prior to the index visit, and about 1/3 of patients (*n* = 15660) had a non-obese BMI measurement in the pre-index visit.

#### Study population characteristics

Table 1 summarizes the study population demographics. Patients were majority male (55.3%). The racial composition was 49.4% White, 34.7% Black or African–American, and 8.8% Hispanic. During the index visit, 57.1% used Private or Commercial insurance and 38.6% used Medicaid/CHIP. At the index visit, 30.5% of patients were 2–4 years old, 42.9% were 5–11 years, and 26.6% were 12–18 years.

**Table 1:** Demographic characteristics of study population.

Indicator	*N* (%)
Gender	
Male	27,503 (55.3%)
Female	22,191 (44.7%)
Race	
Asian	1137 (2.3%)
Black or African–American	17,247 (34.7%)
White	24,562 (49.4%)
Native Hawaiian or Other Pacific Islander	26 (<1%)
American–Indian/Alaska Native	42 (<1%)
Unknown	6043 (12.2%)
Multiple race	637 (1.3%)
Ethnicity	
Hispanic	4360 (8.8%)
Not Hispanic	41,078 (82.7%)
No Information	4038 (8.1%)
Unknown	218 (<1%)
Insurance plan	
Medicaid/CHIP	19,178 (38.6%)
Private/commercial	28,393 (57.1%)
Self-pay	266 (<1%)
Multiple insurance types	840 (1.7%)
No information	1017 (2.0%)
Age at index visit	
2–4 years	15,158 (30.5%)
5–11 years	21,303 (42.9%)
12–18 years	13,233 (26.6%)

#### Visit characteristics

The mean and standard deviation time difference between pre-index and index visits were 303.6 and 462.8 days, respectively, and the median difference was 125 days. The mean and standard deviation time difference between index and post-index visits were 147.8 and 246.3 days, respectively, and the median difference was 49 days. More than two-thirds of clinical observations recorded during pre- and post-index visits were made within 180 days of the index visit (*n* = 129,095) and an additional 13.8% of observations were made between 180 and 365 days of the index visit (*n* = 26,446); over 80% of observations from pre- and post-index visits were made within a year of the index visit. A majority of visits for patients in the study population (90.1%) occurred in an outpatient setting; 8.7% were emergency room visits and 1.2% occurred in an inpatient setting.

### Data analysis

#### Matched control population

To compare clinical condition trajectories between our cohort with obesity and patients with a healthy BMI, a matched control cohort of children with at least one healthy BMI measurement (measurements in the 5th–84th percentiles for age and sex) [20] between 2009 and 2016 and no recorded unhealthy BMI measurements was obtained.

The control patient data selection and cleaning processes are summarized in Fig. 1b. For each visit, patient age and the number of prior visits with a clinical observation in the CHOP system were calculated. The number of prior visits with a recorded clinical observation for each case with obesity prior to the index visit was also calculated. The number of prior visits was intended to serve as a proximate measure of healthcare utilization and clinical well-being.

There were 343,998 eligible patients with 4,936,503 visits with at least one recorded clinical condition. Controls with only one documented visit and visits where patients had an age difference greater than 180 days from the oldest or youngest cases in the study population were excluded. The final control pool consisted of 3,622,341 potential visits and 296,751 patients.

Using the R matchControls function [21], each patient with pediatric obesity was matched with a control patient by sex, number of prior visits, and index age (for the matched control this was the age at the matching visit). Controls were matched by age within 60 days of their matched case. The youngest case patients were matched first. Once a control was matched, all other visits that the patient had in the control pool were removed.

All clinical observations from controls’ matching visits, the visit before, and the visit after (if applicable) were extracted from the PBD database. All controls had a pre-index and index visit (*n* = 49,694) and 89% of controls had a post-index visit (*n* = 44,208).

**Fig. 2:**
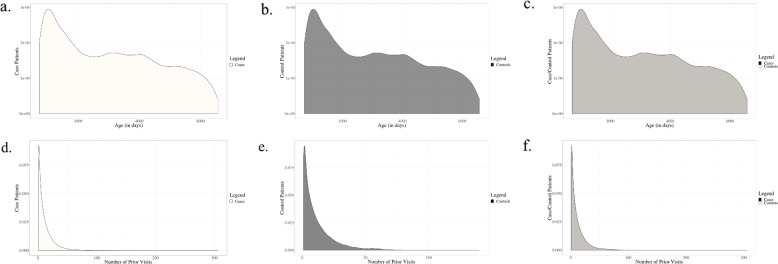
Kernel density estimates (KDE) of the distribution of matched index age and prior healthcare utilization for the case and control populations. **a**, **b** illustrate the distribution of matched index ages for the case and control populations and **c** overlays both distributions on the same axes. **d**, **e** illustrate the distribution of prior healthcare visits for the case and control populations and **f** overlays both distributions on the same axes. Because the distributions are almost identical for the case and control populations, **c** and **f** appear to be a single KDE. The gray color of the overlaid KDEs results from the combined, identical black and white KDEs for the case and control populations.

Figure 2 illustrates the similarity of age distribution and prior healthcare visits among the matched case and control populations. The mean and standard deviation age difference between the matched pair index ages were 0.13 and 1.65 days, respectively, and the median age difference was 0 days. The mean and standard deviation difference in visits prior to the matched index visit were 0.34 and 4.09 visits, respectively, and the median difference was 0 visits. Among control patients, 92.1% of visits were outpatient, 1.2% were inpatient, and 5.2% were emergency room visits. Less than 2% of visits were in other categories, including Administrative or Observation visits.

#### SPADE analysis

The sequential pattern mining algorithm, SPADE [22] was used to mine patient data for temporal patterns in clinical condition trajectories surrounding obesity incidence. SPADE first scans data to identify individual items (e.g., a singular diagnosis in a specific timing class) above a specified support level (e.g., the proportion of patients with an identified condition pattern). Using these frequent single items, SPADE then builds more complex sequences (multiple diagnoses across different timing classes) at the given support level. Thus, a complex sequence present above a given support level is comprised of individual items that also occur above the support level. Prior research has shown that SPADE demonstrates runtime efficiency and low memory usage on sparse datasets [23]. As we were working to identify temporal condition patterns in a large, sparse EHR dataset, we selected SPADE as our pattern mining algorithm for this study.

The R arules package [24] was used to apply SPADE to the clinical data for the cases at a support level of 0.01 (thereby detecting the prevalence of clinical condition sequences present in at least 1% of the cases). The control population data were analyzed to determine prevalence of the patterns found by SPADE in the cases. SPADE analysis executed quickly for our study, with a runtime on the order of single seconds (on a MacBook Pro running MacOS version 10.12.6 and with 8 GB of RAM) using the R implementation. Pairwise McNemar’s tests were used to determine if there was a statistically significant difference in the frequency of these top sequences between the case and control populations. Effect sizes for each sequence were determined by calculating the odds ratios among discordant McNemar’s pairs.

#### Clinical term mapping

There were 7241 unique International Classification of Diseases concept names in the clinical observations for the case population. Many of these conditions were rarely used and, due to this granularity, SPADE would not find the support to detect meaningful condition patterns in the study population. Thus, all clinical observations were grouped into medically homogenous classes using expanded diagnostic clusters (EDCs) from the Adjusted Clinical Group System [8, 25], which places related concepts into fewer groups. Using the Python 3 programming language [26], clinical conditions from both the case and control populations were mapped to 268 unique EDC codes. Some codes had multiple mapped EDC concepts which were all included and treated as distinct conditions; 80% of clinical observations were mapped to 62 EDC codes.

## Results

### SPADE analysis

SPADE identified 189 sequences with a support level of 0.01 or higher among the case population. With clinician input, we removed 12 sequences with conditions that were not clinically informative, including: administrative concerns and nonspecific laboratory abnormalities, other skin disorders, preventive care, and nonspecific signs and symptoms. In addition, we removed 14 sequences with obesity since this diagnosis could only be common in the case population. During their index visit, 7119 patients in the case population (14.3%) received a formal obesity diagnosis.

After removing the 26 sequences with the aforementioned conditions, pairwise McNemar’s tests were administered on the remaining 163 sequences. The McNemar’s tests indicated that 80 sequences had statistically significant (*p* < 0.05) higher levels of support among cases (Table 2) and 45 had statistically significant higher levels of support among controls (Table 3). Although the sequences in Table 3 were initially identified by SPADE as condition trajectories that existed in at least 1% of the case population, they were detected at a significantly higher level of support among the controls. In addition, 23 sequences had statistically insignificant (*p* > 0.05) higher levels of support among cases, and 15 had statistically insignificant higher levels of support among controls.

**Table 2:** Sequences with a statistically significant higher level of support among the case population (*p* < 0.05).

Sequence	Case+/Control+	Case+/Control−	Case−/Control+	Case−/Control−	*p*-value	Effect size (odds ratio)
1-Allergic Rhinitis, 1-Asthma	35	1112	979	47,568	**	1.14
1-Allergic Rhinitis, 1-Asthma, 2-Asthma	14	725	532	48,423	****	1.36
1-Allergic Rhinitis, 2-Allergic Rhinitis	25	1029	782	47,858	****	1.32
1-Allergic Rhinitis, 2-Allergic Rhinitis, 2-Asthma	6	544	353	48,791	****	1.54
1-Allergic Rhinitis, 2-Asthma	25	933	672	48,064	****	1.39
1-Asthma^a^	534	4798	3660	40,702	****	1.31
1-Asthma, 2-Allergic Rhinitis	18	1035	634	48,007	****	1.63
1-Asthma, 2-Allergic Rhinitis, 2-Asthma	14	907	526	48,247	****	1.72
1-Asthma, 2-Asthma^b^	177	2991	1931	44,595	****	1.55
1-Asthma, 2-Dermatitis and eczema	6	566	347	48,775	****	1.63
1-Otitis media, 1-Deafness, hearing loss	19	929	616	48,130	****	1.51
1-Otitis media, 2-Asthma	5	607	319	48,763	****	1.90
1-Deafness, hearing loss	93	2113	1380	46,108	****	1.53
1-Chronic pharyngitis and tonsillitis	17	1089	643	47,945	****	1.69
1-Acute upper respiratory tract infection, 1-Asthma	6	746	505	48,437	****	1.48
1-Acute upper respiratory tract infection, 2-Allergic Rhinitis	8	694	473	48,519	****	1.47
1-Acute upper respiratory tract infection, 2-Asthma	19	1070	641	47,964	****	1.67
1-Acute upper respiratory tract infection, 2-Dermatitis and eczema	6	504	378	48,806	****	1.33
1-ENT disorders, other	7	699	521	48,467	****	1.34
1-Strabismus, amblyopia	6	616	488	48,584	***	1.26
1-Constipation	22	1326	1170	47,176	**	1.13
1-Gastroenteritis	13	889	766	48,026	**	1.16
1-Fever	33	1236	1110	47,315	**	1.11
1-Nausea, vomiting	6	672	473	48,543	****	1.42
1-Nonfungal infections of skin and subcutaneous tissue	10	786	639	48,259	****	1.23
1-Urinary symptoms	21	1226	1023	47,424	****	1.20
1-Headaches	26	930	700	48,038	****	1.33
1-Seizure disorder	15	890	603	48,186	****	1.48
1-Seizure disorder, 2-Seizure disorder	4	606	418	48,666	****	1.45
1-Sleep problems	9	650	402	48,633	****	1.62
1-Autism Spectrum Disorder	6	580	377	48,731	****	1.54
1-Lacerations	2	533	417	48,742	***	1.28
1-Respiratory signs and symptoms	34	1275	1060	47,325	****	1.20
1-Sleep apnea	5	776	277	48,636	****	2.80
1-Contusions and abrasions	24	1285	915	47,470	****	1.40
1-Dermatitis and eczema^b^	156	2726	2493	44,319	**	1.09
1-Dermatitis and eczema, 1-Asthma	8	535	440	48,711	**	1.22
1-Dermatitis and eczema, 2-Asthma	9	593	384	48,708	****	1.54
1-Dermatitis and eczema, 2-Dermatitis and eczema	13	947	720	48,014	****	1.32
1-Exanthems	9	685	522	48,478	****	1.31
1-Dermatophytosis	4	602	511	48,577	**	1.18
2-Allergic Rhinitis^b^	252	3968	2624	42,850	****	1.51
2-Allergic Rhinitis, 2-Asthma	43	1957	978	46,716	****	2.0
2-Allergic Rhinitis, 2-Asthma, 3-Asthma	10	687	493	48,504	****	1.39
2-Allergic Rhinitis, 2-Dermatitis and eczema	5	719	368	48,602	****	1.95
2-Allergic Rhinitis, 3-Asthma	10	791	627	48,266	****	1.26
2-Asthma^a^	679	6215	3602	39,198	****	1.73
2-Asthma, 2-Dermatitis and eczema	15	892	411	48,376	****	2.17
2-Asthma, 3- Allergic Rhinitis	10	695	623	48,366	*	1.12
2-Asthma, 3-Asthma	121	2210	1874	45,489	****	1.18
2-Disorders of lipid metabolism	3	565	220	48,906	****	2.57
2-Chronic pharyngitis and tonsillitis	20	1088	665	47,921	****	1.64
2-Other endocrine disorders	11	971	464	48,248	****	2.09
2-Ophthalmic signs and symptoms	4	555	412	48,723	****	1.35
2-Constipation	44	1657	1210	46,783	****	1.37
2-Gastroesophageal reflux	21	976	757	47,940	****	1.29
2-Urinary symptoms	31	1208	999	47,456	****	1.21
2-Musculoskeletal signs and symptoms	52	1384	1226	47,032	**	1.13
2-Fractures (excluding digits)	82	1713	1514	46,385	***	1.13
2-Bursitis, synovitis, tenosynovitis	4	520	358	48,812	****	1.45
2-Musculoskeletal disorders, other	37	1181	1042	47,434	**	1.13
2-Neurologic signs and symptoms	13	974	675	48,032	****	1.44
2-Headaches	30	888	681	48,095	****	1.30
2-Seizure disorder	16	1076	627	47,975	****	1.72
2-Sleep problems	9	812	388	48,485	****	2.09
2-Developmental disorder	98	2193	1411	45,992	****	1.55
2-Migraines	10	559	362	48,763	****	1.54
2-Autism Spectrum Disorder	17	1035	419	48,223	****	2.47
2-Psychologic signs and symptoms	1	677	405	48,611	****	1.13
2-Psychological disorders of childhood	6	587	327	48,774	****	1.80
2-Respiratory signs and symptoms	45	1320	1089	47,240	****	1.21
2-Sleep apnea	1	734	310	48,649	****	2.37
2-Dermatitis and eczema^b^	201	3215	2270	44,008	****	1.42
2-Acne	33	889	490	48,282	****	1.81
3-Chronic pharyngitis and tonsillitis	4	901	477	48,312	****	1.89
3-Seizure disorder	8	658	549	48,479	**	1.20
3-Sleep problems	2	541	295	48,856	****	1.83
3-Developmental disorder	44	1323	1221	47,106	*	1.08
3-Autism Spectrum Disorder	4	641	402	48,647	****	1.59
3-Sleep apnea	1	557	239	48,897	****	2.33

The numbers before each diagnosis in a sequence represents the diagnosis timing class: “1” denotes that the observation was recorded during a patient’s pre-index visit, “2” represents the index visit, and “3” signifies the post-index visit.

*p < 0.05; **p ≤ 0.01; ***p ≤ 0.001; ****p ≤ 0.0001.

^a^Denotes sequences with support ≥0.1 among cases.

^b^Denotes sequences with support ≥0.05 among cases.

**Table 3:** Sequences with a statistically significant higher level of support among the control population (*p* < 0.05).

Sequence	Case+/Control+	Case+/Control−	Case−/ Control+	Case−/ Control−	*p*-value	Effect size
1-Otitis media, 1-Acute upper respiratory tract infection	22	803	924	47,945	**	1.15
1-Otitis media, 2-Otitis media	89	1408	2117	46,080	****	1.50
1-Otitis media, 2-Otitis media, 3-Otitis media	28	850	1147	47,669	****	1.35
1-Acute upper respiratory tract infection^a^	1141	5680	6766	36,107	****	1.19
1-Acute upper respiratory tract infection, 2-Acute upper respiratory tract infection	51	936	2404	46,303	****	2.57
1-Conjunctivitis, keratitis	20	726	839	48,109	*	1.16
1-Abdominal pain	39	1048	1261	47,346	****	1.20
1-Musculoskeletal signs and symptoms	42	1113	1251	47,288	**	1.12
1-Fractures (excluding digits)	62	1199	1478	46,955	****	1.23
1-Fractures (excluding digits), 2-Fractures (excluding digits)	31	794	1121	47,748	****	1.41
1- Musculoskeletal disorders, other	31	918	1031	47,714	*	1.12
1-Attention deficit disorder	42	766	1273	47,613	****	1.66
1-Acute lower respiratory tract infection	2	551	670	48,471	***	1.22
1-Sinusitis	40	1076	1313	47,265	****	1.22
2-Otitis media^b^	452	2683	4515	42,044	****	1.68
2-Otitis media, 3-Otitis media	86	1305	1954	46,349	****	1.50
2-Deafness, hearing loss	29	1,054	1,336	47,275	****	1.27
2-Acute upper respiratory tract infection^b^	610	2819	6915	39,350	****	2.45
2-Acute upper respiratory tract infection, 3-Acute upper respiratory tract infection	36	569	2251	46,838	****	3.96
2-Conjunctivitis, keratitis	11	543	775	48,365	****	1.43
2-Nonfungal infections of skin and subcutaneous tissue	6	546	633	48,509	*	1.16
2-Abdominal pain	27	1010	1257	47,400	****	1.24
2-Viral syndromes	53	787	1551	47,303	****	1.97
2-Fractures (excluding digits), 3-Fractures (excluding digits)	21	764	939	47,970	****	1.23
2- Developmental Disorder, 3-Developmental Disorder	8	552	623	48,511	*	1.13
2-Cough	27	864	1316	47,487	****	1.52
2-Sinusitis	24	569	1238	47,863	****	2.18
2-Contusions and abrasions	11	644	877	48,162	****	1.36
2-Viral warts and molluscum contagiosum	8	507	580	48,599	*	1.14
3-Allergic rhinitis	111	2039	2546	44,998	****	1.25
3-Otitis media^b^	462	2734	4143	42,355	****	1.52
3-Acute upper respiratory tract infection^b^	594	3229	6421	39,450	****	1.99
3-Fever	14	543	847	48,290	****	1.56
3-Abdominal pain	19	690	1,089	47,896	****	1.58
3-Urinary symptoms	18	760	932	47,984	****	1.23
3-Viral syndromes	42	979	1504	47,169	****	1.54
3-Musculoskeletal signs and symptoms	30	891	1087	47,686	****	1.22
3-Acute sprains and strains	29	815	1011	47,839	****	1.24
3-Fractures (excluding digits)	39	1080	1255	47,320	***	1.16
3-Musculoskeletal disorders, other	11	692	902	48,089	****	1.30
3-Neurologic signs and symptoms	8	522	590	48,574	*	1.13
3-Attention deficit disorder	42	802	1247	47,603	****	1.55
3-Cough	21	804	1240	47,629	****	1.54
3-Sinusitis	17	634	1231	47,812	****	1.94
3-Dermatitis and eczema	75	1779	2123	45,717	****	1.19

The numbers before each diagnosis in a sequence represents the diagnosis timing class: “1” denotes that the observation was recorded during a patient’s pre-index visit, “2” represents the index visit, and “3” signifies the post-index visit.

*p < 0.05; **p ≤ 0.01; ***p ≤ 0.001; ****p ≤ 0.0001.

^a^Denotes sequences with support ≥0.1 among cases.

^b^Denotes sequences with support ≥0.05 among cases.

Table 4 shows the unique EDC codes observed in significant sequences for the case population and control population respectively, as well as shared common conditions. Including obesity diagnoses, there were 40 unique EDC codes represented among statistically significant case sequences and 23 unique EDC codes represented among statistically significant control sequences. There was an overlap of 14 EDC codes between the two groups. These shared conditions can be considered common diagnoses for pediatric patients regardless of obesity status.

**Table 4:** Conditions observed in statistically significant sequences in the case and control populations.

Conditions observed exclusively in significant case sequences	Acne Asthma Autism spectrum disorder Bursitis, synovitis, tenosynovitis Chronic pharyngitis and tonsillitis Constipation Dermatophytosis Disorders of Lipid Metabolism ENT disorders, other Exanthems Gastroenteritis Gastroesophageal reflux Headaches Lacerations Migraines Nausea, vomiting Obesity Ophthalmic signs and symptoms Other endocrine disorders Psychologic signs and symptoms Psychological disorders of childhood Respiratory signs and symptoms Seizure disorder Sleep apnea Sleep problems Strabismus, amblyopia
Conditions observed exclusively in significant control sequences	Abdominal pain Acute lower respiratory condition Acute sprains and strains Attention deficit disorder Conjunctivitis, keratitis Cough Sinusitis Viral syndromes Viral warts and molluscum contagiosum
Shared conditions among significant sequences for cases and controls	Acute upper respiratory tract infection Allergic rhinitis Contusions and Abrasions Deafness, hearing loss Dermatitis and eczema Developmental disorder Fever Fractures (excluding digits) Musculoskeletal disorders, other Musculoskeletal signs and symptoms Neurologic signs and symptoms Nonfungal infections of skin and subcutaneous tissue Otitis Media Urinary symptoms

Conditions unique to significant sequences among the case population included autism spectrum disorder (ASD), sleep apnea, disorders of lipid metabolism, headaches, migraines, and psychological disorders of childhood. EDC codes that were represented among statistically significant sequences for both cases and controls include allergic rhinitis (although this was only common for controls in the post-index visit), otitis media, dermatitis and eczema, fever, acute upper respiratory tract infection, and developmental disorders. Seven conditions were diagnosed exclusively during pre-index visits among patients with obesity: dermatophytosis, ear, nose and throat disorders, exanthems, gastroenteritis, lacerations, nausea/vomiting, and strabismus/amblyopia. No diagnoses were significantly more common during post-index visits alone for the case population.

Asthma was strongly associated with pediatric obesity incidence. The diagnosis was present in 21 unique sequences, and were not present in any sequences with a significantly higher level of support among the control population. Asthma was observed in over 10% of both pre-index visits (*n* = 5332) and index (*n* = 6894) for patients with obesity. Aside from obesity, it was the only condition observed at a support level of 0.1 or higher among the case population.

However, asthma was not as commonly diagnosed during the post-index visit. A diagnosis of asthma without asthmaticus in the post-index visit was present in only three sequences. The sequence with the highest support (2-Asthma, 3-Asthma, indicating asthma diagnoses in the index and post-index visits) was present among 2331 cases, a number markedly lower than the diagnosis of asthma in the pre-index or index visits. A diagnosis of asthma, without status asthmaticus exclusively during the post-index visit (without a prior asthma diagnosis), was not a statistically significant sequence for the case population.

Finally, effect size calculations provided a measure of the strength of associations identified by SPADE. Effect sizes among the significant sequences for the case population ranged from 1.08 to 2.80. Sleep apnea diagnoses across visit timing classes had among the highest effect sizes (2.80, 2.37, and 2.33 for diagnoses during the pre-, index, and post-index visits, respectively). A diagnosis of ASD in the index visit had an effect size of 2.47, indicating a strong association. Asthma diagnosed during the pre-index visit had an effect size of 1.31; the effect size increased to 1.73 for diagnoses during the index visit. Allergic rhinitis diagnoses during the index visit had an effect size of 1.51. Comorbid asthma and allergic rhinitis diagnoses during the index visit had an effect size of 2.0.

## Discussion

### Methodological contributions

Obesity research has typically been formulated using epidemiological approaches wherein an a priori determined hypothesis (e.g., obesity incidence is more prevalent among asthmatics than non-asthmatics) is tested on a particular dataset. Furthermore, most extant obesity research has not considered temporal dependencies between obesity incidence and the occurrence of comorbidities.

One of the key strengths of our study is that it utilized a large, unselected population and the SPADE algorithm to find frequent temporal patterns in clinical data. This approach does not assume an a priori hypothesis regarding the association of obesity with a prespecified covariate. We view this novel data-driven approach as one that complements standard epidemiological methods with the potential to discover important hypotheses for future research and thereby expand our understanding of the complex individual and social factors that affect the obesity epidemic [27, 28]. Using this approach in a retrospective analysis of a pediatric population with obesity, we identified 80 temporal patterns present at statistically significant higher levels than in the matched control population of individuals with only healthy BMI observations. Among these patterns, there were 40 unique condition diagnoses. Seven of these conditions were commonly diagnosed only during pre-index visits and zero were commonly diagnosed only during post-index visits.

### Obesity diagnosis associations

We found strong associations between asthma, allergic rhinitis, and obesity incidence. Although the influence of body weight changes and asthma outcomes requires more exploration, prior research has shown that children who are overweight or obese are more likely to develop asthma [29, 30]. The high prevalence of asthma observed during pre-index visits provides additional evidence in support of the contribution of early-life asthma to pediatric obesity onset [31] and the idea of a bidirectional asthma–obesity relationship. Children with asthma may be particularly susceptible to developing obesity and are targets for intervention efforts. In addition, the lower prevalence of asthma diagnoses during post-index visits suggests that while children with obesity are more likely to develop asthma, there may be a period of time between when children who are newly obese develop the condition.

Prior research is mixed on the relationship between allergic rhinitis and pediatric obesity. Some studies have failed to find a strong association between allergic rhinitis and obesity [32, 33], while Han et al. [34] found reduced odds of allergic rhinitis among children who were centrally obese and Lei et al. [35] found that overweight and obesity actually increased the risk of allergic rhinitis in a pediatric population. In our study, we found that allergic rhinitis was significantly more common among patients with obesity during the pre-index and index visits, but not during the post-index visit. This suggests that further investigation is needed on how body weight changes and BMI trajectory affect allergic rhinitis incidence, and also suggests that children with allergic rhinitis may be more likely to develop an unhealthy body weight. In addition, the comparably high effect size of comorbid asthma and allergic rhinitis during the index visit indicates that asthma may mediate the relationship between allergic rhinitis and pediatric obesity. Further investigation into this potential association is warranted.

Previous studies have indicated that children with intellectual disabilities and ASD have higher rates of obesity than other youth [36, 37]. In our study, developmental disorders (DD) were observed in some sequences that were more common among cases and some sequences that were more common in the control population, but ASD was a common diagnosis during the pre, post, and index visits only for the case population. In addition, DD diagnoses during pre-index visits were not present in significant sequences for either cases or controls. These findings indicate that while there is an association between ASD and obesity, there may be no temporal dependence. Furthermore, there may be differential risk factors for obesity among children with ASD compared with youth with other intellectual disabilities, and children with certain DD outside of ASD may be more at risk for developing obesity than others. Further investigation into these risk factors as well as the temporality trends in DD diagnosis and obesity incidence observed in this study is necessary.

### Obesity diagnosis using EHR data

Although all patients in the case population had an obese BMI measurement during their index visit, only a fraction (approximately one in seven) received a formal obesity diagnosis. Pediatric obesity remains underdiagnosed in clinical practice, and children who are overweight or obese lack comprehensive access to nutrition and physical activity counseling [38, 39]. Unhealthy BMI identification and documentation improves clinical weight management [40, 41]. While low physician diagnosis of child overweight and obesity is well documented, past studies that investigated EHR use to address childhood obesity in a clinical context have relied on retrospective chart review [40, 42, 43], clinician surveys [44], or mixed methods of surveys and patient record review [45], and utilized prevalence estimates of overweight and obesity in a pediatric population to calculate diagnosis. In contrast, our study employs EHR data to characterize clinical weight management at the time a child’s BMI first was classified as obese, and provides critical support for integrating recommendations from clinical practice guidelines regarding childhood obesity directly into EHR systems (such as BMI alerts) and scaling up weight management and education in pediatric clinical care settings.

### Limitations of the study

Our findings are descriptive and the discovered temporal patterns and comorbidities should be viewed in this light. No causality can be attributed to the associations uncovered in this study. In addition, a greater proportion of our controls had a post-index visit than the cases which may have affected the associations in sequential patterns with conditions recorded during the post-index visit. Potential explanations include pure chance in the matching process or that vulnerable children are more likely to become obese and may face greater barriers to obtaining healthcare. These disparities may manifest in fewer primary care visits, which may explain the lower proportion of children with obesity who had a post-index visit. Another limitation is that approximately one-third of study cases had no BMI measurements in the EHR prior to the index visit. Assuming some of these individuals were obese prior to the index visit could imply a reduction in support for sequences with conditions in pre-index and index visit and shift to sequences with those conditions in the index and post-index visit. A final limitation to the study lies in the data itself. Relying on diagnostic codes within EHRs may lead to an underdiagnosis of certain conditions (which contributed to the use of BMI *z*-score measurements instead of a formal medical diagnosis of “obesity” in this study) [46, 47]. However, resolution of this concern is likely condition dependent and could involve complex methodology that was outside the scope of this study. However, assuming underdiagnosis rates are similar between cases and controls, which we expect given that healthcare utilization was a criterion for matching, we anticipate that the effect of underdiagnosis would be a decrease in sensitivity. That is, our methodology may fail to discover some significant patterns in the presence of underdiagnosis, but the patterns that are discovered will retain the specified support level and should retain the same level differences between the cases and controls.

### Future work

This study revealed key areas of future investigation. Associations between pediatric obesity incidence and comorbidities including asthma and allergic rhinitis should be further investigated to uncover potential causal relationships, as should unique and differential causal risk factors for obesity among children with ASD and other DD. In addition, unique causal risk factors for obesity among patients with conditions only associated with obesity in the pre-index visit should be investigated. Future work can also examine the effect of mediating factors such as demographic and socioeconomic indicators on the uncovered associations. Finally, the low rates of formal documentation of obesity in patients’ EHRs identified in this study suggest the need for improved clinician education on the importance of obesity diagnosis and implementation of pediatric weight management guidelines. Future research should focus on understanding optimal methods for integrating pediatric weight management clinical decision support tools into EHR systems and promoting clinical adherence to pediatric weight management guidelines.

## Data Availability

The code used for data acquisition, processing, and analysis in this study may be found at: https://github.com/chop-dbhi/masino-lab-obesity-incidence.
